# Dog Ecology and Population Studies in Lagos State, Nigeria

**DOI:** 10.5539/gjhs.v6n2p209

**Published:** 2014-02-14

**Authors:** Sunday E. Hambolu, Asabe A. Dzikwi, Jacob K.P. Kwaga, Haruna M. Kazeem, Jarlath U. Umoh, Dupe A. Hambolu

**Affiliations:** 1 Department of Veterinary Public Health and Preventive Medicine, Faculty of Veterinary Medicine, Ahmadu Bello University, Zaria, Kaduna State, Nigeria; 2 Department of Veterinary Microbiology, Faculty of Veterinary Medicine, Ahmadu Bello University, Zaria, Kaduna State, Nigeria; 3 Federal Department of Livestock and Pest Control Services, Federal Ministry of Agriculture and Water Resources, Abuja, Nigeria.

**Keywords:** dog, ecology, Lagos, Nigeria, rabies, vaccination

## Abstract

Dog population dynamics have a major impact upon the effectiveness of rabies control strategies. As such, understanding domestic dog ecology has been recognized as central to the design of effective rabies control programmes. This study was conducted to determine the dog ecology in Lagos State using compound dog count and street dog count in the three senatorial districts (Lagos West, East and Central) of Lagos State from February, 2011 to January, 2012. A total of 546 questionnaires were distributed for the compound dog count and all were completed and returned. Various aspects of dog ecology were determined, including size, sex, breed of the dog population, management of dogs and rabies awareness among the respondents. Out of the 546 compounds surveyed, 518 (94.87%) owned at least one dog. A total of 1,427 dogs were counted from the street counts while a total of 1,447 dogs (2.8 dogs/compound) were counted from the compound count. The dogs comprised of 583 males and 864 females, out of which 64.10% are confined. The dog vaccination coverage in the dog population surveyed was 64.10% and administered majorly (91.30%) by veterinarians. Security (60%) and pets (26%) were the major reasons for keeping dogs. Majority (88.80%) of the respondents were aware of rabies and its mode of transmission, but still believed in the use of concoctions (40.40%), herbs (19.90%) and consumption of the organ of the offending dog (11.50%) for the treatment of rabies. The findings of this study showed a male: female ratio of dog to be 1:1.5 and a dog: human ratio of 1:5.6. There was also a responsible dog ownership as majority of the respondents do confine, vaccinate and provide food for their dogs. Vaccination coverage of the total dog population was however below the 70-80% target recommended by the World Health Organization to achieve herd immunity.

## 1. Introduction

Dog population dynamics have a major impact upon the effectiveness of rabies control strategies. An understanding of domestic dog ecology has been recognized as central to the design of effective rabies control programmes (Cleaveland et al., 2006; Hsu et al., 2003; Slater, 2001).

The human population of Africa expanded enormously during the 20^th^ century, and the dog population expanded in parallel (Brooks, 1990). In Africa, dogs are intimately dependent on humans for food and shelter (Brooks, 1990), and this association means that dog populations can be correlated, in size as well as distribution with human populations. Social change, such as urbanization, resulted in an increase in human and dog movement as well as interactions between domestic and stray dogs. Domestic dogs are the main reservoir of rabies throughout the developing world (Arai et al., 2001). This may have enhanced persistence of the rabies virus in the local dog population and greater movement of infected dogs (Bingham, 2005). More than 90% of all human deaths from rabies occur in the developing world (WHO, 1998). In Africa and Asia, an estimated 24,000-70,000 people die of rabies each year (Knobel et al., 2005) and the domestic dog is the main source of exposure and primary vector for this important human disease (Wandelar et al., 1993).

Rabies epidemiology is highly dependent and responsive to human and dog-population densities as well as to the cultural and socioeconomic environment in which the two populations interact (De Mattos et al., 1999). Rabies has been neglected in several African countries and the major constraints to its effective control are economic and logistic, rather than technical, with poor infrastructure and inadequate resources hampering control programs (Perry, 1993). Rates of disease transmission depend on the density of the dog population and social behavior that determines the extent of contact (Ratsitorahina et al. 2009). Studies of dog ecology and demography have been conducted in different parts of the world (Brooks, 1990; Kitala et al., 2001; Flores-Ibarra & Estalla-Valenzuela, 2004; Kongkaew et al., 2004; Hossain et al., 2013). Despite the fact that rabies is reported to be endemic in Nigeria, data on dog ecology including their population densities, population structure and characteristics in different state of Nigeria are scares. In Nigeria, there have been only a few studies that have tried to estimate the dog population size in urban or rural areas; a demography and dog ecology study was last conducted in Lagos state about 24 years ago by Oboegbulem & Nwakonobi (1989) which revealed a dog:human ration of 1:21 and 1:45 in Lagos Urban and rural respectively. Another study was also carried out in 1984 by Ezeokoli et al. in Northern and Southern Kaduna which revealed a dog:human ratio of 1:3 and 1:27 respectively, however no recent studies has reported the demography of domestic dogs in Lagos state to the best of our knowledge. This scarcity together with the lack of political will and systemic corruption are a major obstacle for the implementation of a national rabies control program population.

Lagos state which is both a political and economic importance in Nigeria has a highly dense human population due to the movement of people from neighbouring states in the country as well as other parts of the world into the state. Since, an increase in human population is related to an increase in dog population due to their need as security, pets and for hunting purposes. The area provides an ideal condition for the maintenance of a high and dense population of susceptible dogs that have the potential to act as reservoirs for the maintenance and transmission of canine rabies. Therefore, knowledge of the dog ecology and demography will fill an important logistical gap in rabies control and in understanding the real threat of such pathogens, in achieving effective disease control, and in managing dog population growthin Lagos state in particular and Nigeria as a whole. The results study might be applicable to developing dog-control measures for effective rabies control, and for predicting the needs of dog-vaccination programs to prevent canine rabies especially during yearly World Rabies day vaccination campaigns. Also, comparing the results estimates based on human:dog ratios will give us a likely indication of the from other regions provides an evaluation of the appropriateness of that practice. Therefore, this study was conducted to determine the dog ecology in Lagos State using compound and street counts.

## 2. Materials & Methods

### 2.1 Study Site

The study was conducted in Lagos state, Nigeria. The state is the most dense and second most populated in the country with about 9 million people (Census, 2006). The State is located on latitude 6°2´N to 6°4´N and longitude 2°45´E to 4°20´E and is made up of 20 Local Government Areas comprising three senatorial districts i.e. Lagos East, Lagos West and Lagos Central senatorial districts. It is a costal State bounded by Ogun state to the north and east, Atlantic Ocean to the south and the Republic of Benin and Togo to the west.

### 2.2 Survey Approach

The study was designed as a cross-sectional study and was conducted between February, 2011 and January, 2012 using a designed compound questionnaire and the street count. For the dog ecology and street counts, four wards in Lagos West senatorial district and two wards each of Lagos East and Central senatorial districts were randomly selected. Ten streets were selected at random in each of the selected wards and questionnaire for the compound survey of dogs were administered to every fifth house on each of these streets. The street count was carried out early in the morning between 6.00am and 7.30am, a time of maximum dog activity, less human activity and good visibility (Okoh, 1986). Special areas such as specific markets, dump sites, abattoirs/slaughter slabs, and industrial waste disposal sites that are best for observation of stray dogs were given special attention during the street dog count. An estimate of the entire dog population on the street was determined from all street counts in the three senatorial districts using the method and formula described by Beck (1984);

N=∑ (Mn)/∑m

Where;

M = the number of dogs photographed each time and considered marked (observed).

m= the number of dogs recognised as previously observed

∑m = the summation of m to that point in time

n= total number of dogs previously observed i.e each day’s observation (M) less those previously observed (m) would be added to each day

Mn = the product of each day’s M and n

∑(Mn) = the summation of Mn to that point in time, and

N = the population estimate.

For the compound count a pretested questionnaire consisting of three parts was used for the study;

1) Information about compounds and dog population studies e.g. no. of dogs owned, ages of the dogs, breed of dogs, etc.
2) Management and care of dogs e.g. who feeds the dogs, are the dogs confined or not, vaccination status, etc.
3) Cases of dog bites, post exposure management and sequel e.g. has there been any case of dog bite, who owns the offending dog, was treatment sought after the bite, etc.

The same streets used during the street count were also used for this aspect. Every fifth house was selected beginning from the first house on each selected street. An adult member in each of such fifth compound on each side of the selected street was interviewed for about 5-10 minutes using the questionnaire.

### 2.3 Data Analyses

Data obtained were analysed using Chi square to test for association of variables obtained from questionnaires with the aid of the Statistical Package for Social Sciences (SPSS) Version 17.0 and the result presented in form of charts and tables.

## 3. Results

### 3.1 Compound and Street Counts of Dogs

A total of 546 compounds in the three senatorial districts were interviewed for the compound count out of which 518 compounds (94.87%) owned a total of 1,447 dogs. During the street count, a total of 1,427 dogs were counted while during the compound count a total of 1,447 dogs were counted (Table 1).

**Table 1:** Street and compound counts of dogs in the three senatorial districts of Lagos State

Senatorial district	Street count averageno. of dogs	No of dogs from compound counts
Lagos East	295	254
Lagos Central	370	361
Lagos West	762	832
Total	1427	1447

The total number of individuals in the 546 compounds was 8,081 with an average of 14.8 individuals in each compound. There was an average of 179 dogs per 1000 individuals and a dog: human ratio of 1:5.6 (Table 2).

**Table 2:** Structure of dog and human population in the compound count in three senatorial districts of Lagos State

Variables	Frequency
No of compounds sampled	546
Total number of people	8081
Total number of dogs	1447
Dogs per compound	2.8
Dogs per 1000 persons	179
Dog: Human ratio	1:5.6

Estimated dog population in Lagos state = 1,527,718.

Estimated dog population in Lagos state = 1,527,718.

The human population of Lagos State from the 2006 census conducted by the National Population Commission was 9,013,534. Therefore, the total dog population in Lagos State from the dog: human ratio of 1:5.6 obtained in this study, when extrapolated is 1,527,718.

### 3.2 Demography of Dog Population

The age structure of the dogs owned by the 514 respondents is displayed in table 3. Thirty-one percent (459) of the dogs were young, a year old or less, while about 68.28% (988) were above one year of age. There was a predominance of female dogs (59.71%) compared to male dogs (40.29%) with a sex ratio (male:female) of 1:1.5 and majority of the dogs were local breeds (41.9%), 29.5% crossbreeds and 25.5% exotic breeds (Table 3).

**Table 3:** Demographic structure of the dogs kept by the respondents in compound count in the three senatorial districts of Lagos State

Variable	Frequency (%)
**Sex**	
Males	583 (40.29)
Females	864 (59.71)
Total (male:female ratio)	1,447 (1:1.5)
**Age group**	
Young (< 1)	459 (31.72)
Adult/matured (>1)	988 (68.28)
Total	1,447
**Breed of dogs**	
Exotic	427 (29.50)
Crossbreed (Mongrel)	414 (28.60)
Local	505 (41.90)
Total	1,447

### 3.3 Dog Management and Handling Practices

Majority (94.40%) of the respondents do not allow stray dogs access into their compounds, confined (64.09%) and vaccinated (64.10%) their dogs. Veterinarians were responsible for 91.30% of the vaccination, veterinary health assistants (5.10%) and 1.20% of the respondents vaccinated their dogs themselves (Table 4). 113(34.10%) of the respondents vaccinate their dogs yearly, 190 (57.20%) vaccinated as advised by the veterinarian and 29 (8.90%) vaccinate their dogs at their convenience. Cost of vaccination was responsible for not vaccinating by 23.70% of the respondents, not aware (4.30%), and not necessary (26.90%) (Table 4).

**Table 4:** Attitude of respondents towards vaccination of their dogs

Variables	Frequency for responses (%)
**Allow stray dogs into their compounds**	
Yes	31 (5.60)
No	515 (94.40%)
Total	446
**Vaccinate their dogs**	
Yes	332 (64.10)
No	186 (35.90)
Total	518
**Vaccinator of the dogs**	
Veterinarian	303 (91.30)
Animal Health Assistants	12 (5.10)
Self	4 (1.20)
Self and Veterinarian	8 (2.40)
Total	332
**Frequency of dog vaccination**	
Yearly	113 (34.10)
As advised by Veterinarian	190 (57.20)
At convenience	29 (8.90)
Total	332
**Reason for not vaccinating dogs**	
Not necessary	50 (26.90)
Cost	44 (23.70)
No	84 (45.20)
reason Not aware of need to vaccinate	8 (4.3)
Total	86

There was a statistically significant association between confinement and vaccination of dogs (Table 5).

**Table 5:** Attitude of dog owners encountered during a compound count of dogs in Lagos state to confinement and vaccination of their dogs

Variables	Total number of households (%)	Vaccinate their dogs (%)	*p value*
**Restriction status of dogs**			< 0.05
Confined	332 (64.09)	300 (90.36)	
Not confined	186 (35.91)	32 (17.20)	
Total	518	332	

**Figure 1:**
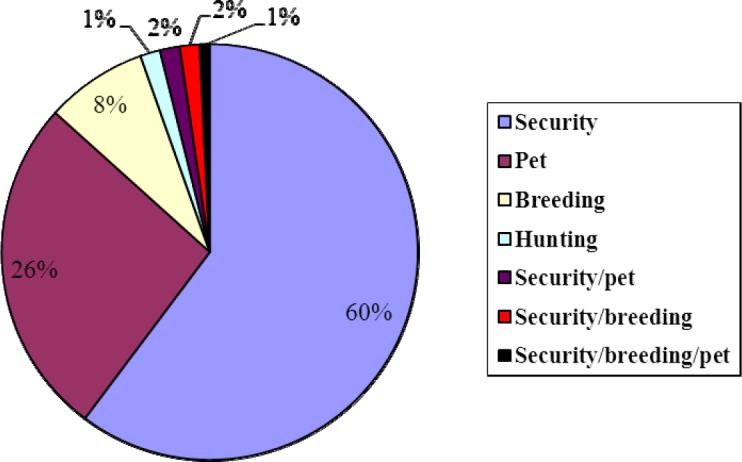
Function of dogs owned by the respondents in the compounds surveyed

Majority (43.63%) of the dogs were maintained on leftover foods and waste, 25.68% on commercial food and 2.3% left them to scavenge for food. The most common source of dogs was purchase from breeders (49.80%), 39.00% as gifts, 0.8% purchased from the market and 10% inherited their dogs. On the mode of depopulation of dogs, 63.89% sell them off, 26.06% give out as gifts, 7.30% slaughter for consumption while 2.32% euthanize them (Table 6). Security was the primary function of majority (60%) of the dogs, 26% kept them as pets, 8% kept them for breeding while for 6% they have a combined function (Figure 1).

**Table 6:** Type of feed, source of dogs and means of dog depopulation in Lagos State

Variables	Frequency of respondents (%)
**Type of feed given to dogs**	
Fed on commercial feed	133 (25.7)
Cook special food	125 (24.1)
fed on family left over	226 (43.6)
Cook/commercial feed	22 (4.24)
Scavenge	12 (2.32)
Total	518
**Source of the dogs**	
Purchased from breeders	256 (49.8)
Purchased from the market	4 (0.8)
Obtained as a Gift	202 (39.0)
Inherited	52 (10)
Gift/purchased from the market	4 (0.8)
Total	518
**Methods of dog depopulation**	
Sell off	331 (63.89)
Give out as gift	135 (26.06)
Slaughter	40 (7.73)
Euthanize	12 (2.32)
Total	518

### 3.4 Demographic Features and Knowledge of Rabies among the Respondents

There was a statistically significant association (*p<0.05*) between age, and awareness of rabies, with majority (260, 47.98%) of the respondents between the ages of 21 and 30 years old, however, there was no statistically significant association between sex and awareness of rabies 66.48% of them males (Table 7) and 37% of them females (Table 7).

**Table 7:** Demographic features of dog owners and association between age, sex of respondents and awareness to rabies in three senatorial districts of Lagos State

Variables	Total number of respondents (%)	Awareness (%)	*P value*
**Age groups (yrs)**			
≤ 20	21 (3.85)	17 (3.1)	0.0001
21– 30	260 (47.98)	242 (44.3)	
31– 40	130 (23.31)	125 (22.9)	
41 – 50	109 (19.96)	81 (14.8)	
>50	28 (4.40)	20 (3.7)	
**Total**	**546**	**485**	
**Sex**			0.2496
Males	363 (66.48)	318 (58.2)	
Females	183 (33.52)	167 (30.6)	
**Total**	**546**	**485**	

**Figure 2:**
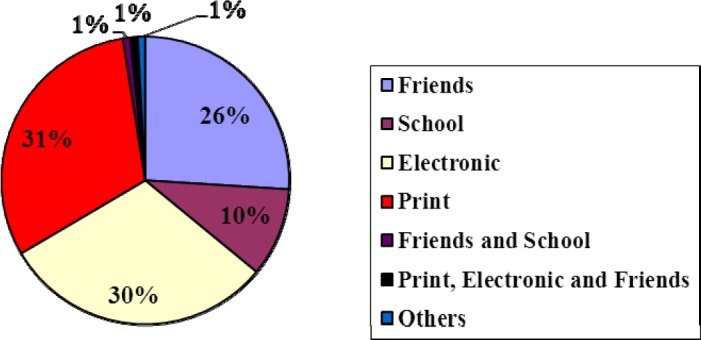
Source of information on rabies among the respondents in three senatorial districts of Lagos State

The most-common sources of information on rabies available to the respondents were print media (31%), electronic media (30%), schools (10%) and friends (26%). One percent of the respondents each obtained their information from a combination of friends and school, print/electronic/friends and other sources (Figure 2).

Dog bite was identified by majority (90.9%) of the respondents as the major route of rabies transmission while 7.4% did not know the mode of transmission. Furthermore out of the 445 respondents 196 (40.4%) said concoctions are used for treating rabies, 96 (19.9%) said herbs can be used, 56 (11.5%) said consumption of offending dog tissue/organ, while 137 (28.2%) said they did not know (Table 8).

**Table 8:** Mode of transmission, name for rabies in the local dialect of the respondents and mode of management of dog bites among the respondents in three senatorial districts of Lagos State

Variables	Response (%)
**Means through which rabies is transmitted**	
Dog bite	441 (90.9)
Playing with dogs	4 (0.8)
Scratch, bite and play with dogs	4 (0.8)
Don’t know	36 (7.4)
Total	485
**Name of rabies in their language**	
Mad dog	278 (57.3)
Rabies	28 (5.8)
Don’t know	179 (36.9)
Total	485
**Traditional way of treating rabies**	
Concoction	196 (40.4)
Herbs	96 (19.9)
Consumption of offending dog tissue/organ	56 (11.5)
Don’t know any traditional method i.e are only aware of treatment in hospitals	137 (28.2)
Total	485

### 3.5 Rabies Exposure among Members of the Compounds Surveyed

Members of 44 (8.1%) out of the 546 compounds had been bitten by a dog, and 81.80% (36/44) of these had received post-exposure treatment. Of the 44 compounds, 24 reported that the treatment was administered by a nurse, 12 by a physician and 8 said they did not know the qualification of the individual that administered the post-exposure treatment. Among the victims, 8 (18.20%) were bitten by their own dogs or dogs owned by someone else i.e neighbours (81.80%) and both victim and the offending dog were apparently healthy (Table 9).

**Table 9:** Dog bite management among respondents in dog-owning compounds surveyed in Lagos State

Variables	Respondents (%)
**Anybody bitten by a dog in the compound**	
Yes	44 (8.1)
No	474 (91.9)
Total	518
**Owner of offending dog**	
Neighbour	36 (81.8)
My own dog	8 (18.2)
Unknown dog	0
Total	44
**Status of the dog after the bite**	
Healthy	44 (100)
Died	0
Sick	0
Total	44
**Status of the victim after bite**	
Healthy	44 (100)
Died	0
Sick	0
Total	44
**If treatment was administered after bite of the victim**	
Yes	36 (81.8)
No	8 (18.2)
Total	44
**Who administered the treatment**	
Nurse	24 (66.7)
Physician	12 (33.3)
Traditional	0
Total	36

## 4. Discussion

Knowledge of dog ecology and dog population demographics is essential in planning effective mass vaccination campaigns and controlling rabies in dogs (Cleaveland et al., 2006). Domestic dogs are the main reservoir of rabies throughout the developing world. As such, understanding domestic dog ecology has been recognised as central to the design of effective rabies control programmes (Beran 1982; Beran & Frith, 1988; Kitala & McDermott, 1995; Wandeler, 1985; Wandeler et al., 1993; WHO, 1987; WHO/WSPA, 1990). In Africa, dogs are heavily dependent on humans for food and shelter, so the sizes of dog populations can be correlated with the human population (Matter & Daniel, 2000). The dog:human ratio (1:5.6) obtained in this study is higher than that reported by Oboegbulem & Nwakonobi (1989) in Lagos Urban (1:21), and in Lagos rural (1:45) and that reported by Ezeokoli et al. (1984) in Southern Kaduna (1:27) but though consistent with what has been reported by Ezeokoli et al. (1984) in Northern Kaduna (1:3) and those of other studies elsewhere in the world: dog;human ratios of 1:8 in Kenya (Kitala et al., 2001), 1:4.5 in Zimbabwe (Brooks, 1990), 1:4.3 in Mexicali (Flores-Ibarra & Estalla-Valenzuela, 2004), and 1:4.6 in Thailand (Kongkaew et al., 2004). This variation may have resulted from the increased human population and the need for dogs for security purposes in the face of the increasing security challenges. In spite of this high dog: human ratio only about 64.1% of respondents vaccinated their dogs against rabies suggesting that only about 64% of the dogs in Lagos State are likely vaccinated against rabies. This is below the WHO recommended level which is needed for effective control of rabies in a dog population. The critical percentage of dogs needed to be immunized to prevent or control an outbreak of rabies is estimated to be 70% (WHO, 1992; Coleman & Dye, 1996). As also suggested by Fekadu (1991), vaccination coverage of at least 80% may be necessary to break transmission of rabies in situations where stray and ownerless dogs predominate. Though, if the vaccination coverage is not maintained, a sufficient susceptible population rebuilds and rabies is re-established rapidly (Beran, 1991; Cleaveland *et al.*, 2006). Mass vaccination of between 60% and 70% of owned dogs has subsequently resulted in dramatic decreases in the incidence of human rabies (Cleaveland *et al.*, 2006).

The high level of poverty among the populace affects their ability to feed their dogs which may have accounted for the high population of stray/roaming dogs (as seen from the street count of dogs which almost equalled the compound count) that rely on the garbage sites spread around the state. This high level of free roaming dogs bring them into close physical contact other dogs and humans i.e. increase dog-dog or dog-human contact rates, thereby increasing the transmission intensity and spread of rabies or other zoonotic diseases present in the population. They may also serve as source of environmental contamination for zoonotic helminth parasites as well as a public health threat through physical injuries to humans through their bites, a bite which may be malicious or due to defensive aggression (protection of territory and resources including resting places, offsprings, food and caregivers).

Also the population density of Lagos state which is a major city in Nigeria and Africa and its location (bordering other countries in Africa) makes it a high priority area for achieving not just high levels of rabies vaccination coverage but also sustained levels. As this large population of dogs may become an established foci (that is challenging to control) for the maintenance and spread of zoonotic and other canine diseases to neighbouring states (Ogun), countries (Republic of Benin and Togo), bordering Lagos state as well as to other parts of the world.

One of the important implication of the above findings (high dog density, high dog: human ratio and the high proportion of likely unvaccinated dogs based on the response of the respondents) and its consequences on the transmission and maintenance of rabies in the study area is that there is a large population of susceptible dogs which may serve as reservoir provide an ideal condition for the spread of the rabies virus to both the dog and human population.

The sex ratio of dogs in this study is skewed towards female dogs i.e. female dogs were more prevalent than male dogs in Lagos State. This is in agreement with the findings of Oboegulem & Nwakonobi (1989) in Lagos urban (0.9:1) and Lagos rural (0.8:1). However, this does not agree with the results of other workers from other parts of the world (Beran, 1982; Daniels &Bekoff, 1989; Brooks, 1990; Cleaveland, 1996; Butler & Bingham, 2000; Kitala et al., 2001; Flores- Ibarra & Estrella-Valenzuela, 2004; Kongkaew et al., 2004). This may indicate that in Lagos State, as compared to other parts of the world, there is a preference for female over male dogs. This preference may be connected with the use of dogs for breeding purposes and also as pets because of their docile nature. Some individuals keep dogs as a source of income, when the dog whelp the offsprings are sold. Though majority (> 60%) of the respondents stated that security is primary function of their dogs and male dogs are mostly used in many parts of the world for this purpose, some individuals still prefer female dogs for this purpose as male dogs are believed to have the tendency of wandering about in search of mating partners (Kitala et al., 2001). The skewed female population has both negative and positive implication for rabies control. This is because the high female populationmay be important in the reduction of rabies spread as it reduces the tendency for more males to aggregate around one particular female thus reducing the aggression and fights that ensue during such contests and thus reducing the spread of rabies among the dog population. Another implication of the high female population (though the reproductive status (spayed or not) was not considered in this study) is the potential for a large population of dogs in the near future which might likely produce negative consequences for the rabies control programmes.

Guard duties have also been identified as the primary reason for keeping dogs in a number of states in Nigeria (Oboegbulem & Nwakonobi, 1989), other countries of Africa such as: Zimbabwe (Brooks, 1990), Zambia (De Balogh et al., 1993) and other parts of the world (Rangel et al., 1981). The needs for guarding property and protecting the compounds in the face of the increasing security challenges in Nigeria may also explain the high human: dog ratio found in this study.

The majority of the people interviewed were skilled (84%) and are engaged in income generation activities whereas 16% are students. This indicates that most dog owners have a source of income and have the capacity to feed their animals which is, reflected in the management of dogs as most (50.04%) people were found to budget for their animals and special diet is provided for them. This may be also responsible for low percentage (5.6%) of dogs that stray around and the high number (64.1%) of compounds that vaccinate their dogs. This is in agreement with the findings of Wise and Kushman (1984) who indicated a relationship between dog ownership and compounds income.

Most of the respondents had some knowledge about rabies and vaccinated their dogs against rabies suggesting they can easily appreciate messages provided during rabies campaigns and will be willing to participate in such campaigns. Despite this knowledge and awareness of rabies, there is the problem of inadequate knowledge of the management protocols of the disease as most of the respondents still believe in the use of concoction, herbs or consumption of the organs of the offending dogs. This has a serious implication on the control and prevention of rabies in the country.

## 5. Conclusions and Recommendation

The results of this study indicate a high density of dogs in Lagos State with a dog to human ratio of 1:5.6., and an average of 2.8 dogs per compound. There were more females (male: female ratio = 1:1.5) compared to male dogs and the age structure tilted towards matured dogs (>1 year of age). There also appears to be a responsible dog ownership among dog owners in Lagos State as majority of them were found to confine, vaccinate and provide feed for their dogs, though the vaccination coverage is below the 70-80% coverage recommended by the World Health Organization.

There is need for the State and Local Government authorities to put in place regular and compulsory vaccination of dogs especially against rabies so as to be able to achieve the recommended 70-80% total dog vaccination coverage level and thus prevent and control the spread of rabies. This can be achieved through the use of enlightenment campaigns in the print, electronic media and a community participatory awareness.
